# Clinical impact of ^18^F-FDG-PET among memory clinic patients with uncertain diagnosis

**DOI:** 10.1007/s00259-020-04969-7

**Published:** 2020-07-31

**Authors:** Giulia Perini, Elena Rodriguez-Vieitez, Ahmadul Kadir, Arianna Sala, Irina Savitcheva, Agneta Nordberg

**Affiliations:** 1 Department of Neurobiology, Care Sciences and Society, Division of Clinical Geriatrics, Center for Alzheimer Research, Karolinska Institutet, 141 52 Stockholm, Sweden; 2 Center for Cognitive and Behavioral Disorders, IRCCS Mondino Foundation and Dept of Brain and Behavior, University of Pavia, 27100 Pavia, Italy; 3 Theme Aging, The Aging Brain Unit, Karolinska University Hospital, 141 86 Stockholm, Sweden; 4 Medical Radiation Physics and Nuclear Medicine Imaging, Section for Nuclear Medicine, Karolinska University Hospital, Stockholm, Sweden

**Keywords:** ^18^F-Fluorodeoxyglucose-PET, Clinical impact, Incremental diagnostic value, Dementia, Mild cognitive impairment

## Abstract

**Purpose:**

To assess the clinical impact and incremental diagnostic value of ^18^F-fluorodeoxyglucose (FDG-PET) among memory clinic patients with uncertain diagnosis.

**Methods:**

The study population consisted of 277 patients who, despite extensive baseline cognitive assessment, MRI, and CSF analyses, had an uncertain diagnosis of mild cognitive impairment (MCI) (*n* = 177) or dementia (*n* = 100). After baseline diagnosis, each patient underwent an FDG-PET, followed by a post-FDG-PET diagnosis formulation. We evaluated (i) the change in diagnosis (baseline vs. post-FDG-PET), (ii) the change in diagnostic accuracy when comparing each baseline and post-FDG-PET diagnosis to a long-term follow-up (3.6 ± 1.8 years) diagnosis used as reference, and (iii) comparative FDG-PET performance testing in MCI and dementia conditions.

**Results:**

FDG-PET led to a change in diagnosis in 86 of 277 (31%) patients, in particular in 57 of 177 (32%) MCI and in 29 of 100 (29%) dementia patients. Diagnostic change was greater than two-fold in the sub-sample of cases with dementia “of unclear etiology” (change in diagnosis in 20 of 32 (63%) patients). In the dementia group, after results of FDG-PET, diagnostic accuracy improved from 77 to 90% in Alzheimer’s disease (AD) and from 85 to 94% in frontotemporal lobar degeneration (FTLD) patients (*p* < 0.01). FDG-PET performed better in dementia than in MCI (positive likelihood ratios >5 and < 5, respectively).

**Conclusion:**

Within a selected clinical population, FDG-PET has a significant clinical impact, both in early and differential diagnosis of uncertain dementia. FDG-PET provides significant incremental value to detect AD and FTLD over a clinical diagnosis of uncertain dementia.

**Electronic supplementary material:**

The online version of this article (10.1007/s00259-020-04969-7) contains supplementary material, which is available to authorized users.

## Introduction

Accumulating evidence for highly specific patterns of hypometabolism in different dementia diseases has led ^18^F-fluorodeoxyglucose PET (FDG-PET) to be regarded as a supportive feature in multiple clinical/research diagnostic criteria [1–5]. The European Association of Nuclear Medicine and the European Academy of Neurology (EANM-EAN) have recently recommended the use of FDG-PET both in dementia [6, 7], when the clinical picture and structural imaging are “unclear,” and in mild cognitive impairment (MCI) [7, 8]. In MCI, the use of FDG-PET has been recommended particularly due to its high negative predictive value and despite limitations due to its substantial variability in detecting MCI due to Alzheimer’s disease (AD) and the limited knowledge on its accuracy to predict conversion to non-AD dementia.

Evidence-based assessment of the clinical utility of FDG-PET is challenging, and the incremental diagnostic value of FDG-PET versus a clinical diagnosis is not well known [9]. Head-to-head comparison between baseline clinical diagnosis and post-FDG-PET diagnosis versus a common reference standard is critical to quantify the incremental diagnostic value of FDG-PET, but these types of studies are lacking [9]. As pathophysiological biomarkers are increasingly incorporated in clinical evaluation, testing the utility of FDG-PET versus a baseline diagnosis that incorporates cerebrospinal fluid (CSF) analyses is of utmost importance.

In this study, we evaluate the utility of FDG-PET among memory clinic patients who, after extensive cognitive assessment, received an uncertain diagnosis, in terms of (1) change in diagnosis from baseline to post-FDG-PET and (2) change in diagnostic accuracy when comparing each baseline and post-FDG-PET diagnoses to a long-term follow-up diagnosis used as reference, and (3) FDG-PET performance testing in MCI and dementia patients.

## Methods

### Study design and participants

The study was designed as a single-site cross-sectional study with a delayed verification, to assess the clinical diagnostic utility of FDG-PET in a selected population of cognitively impaired patients with an uncertain diagnosis who met appropriate use criteria for clinical FDG-PET. The study population consisted of 277 patients attending the Clinic for Cognitive Disorders, Theme Aging, Karolinska University Hospital, Stockholm, Sweden. Patients were seen between 2012 and 2014 and had been mainly referred by primary care physicians (GPs), but also from different hospital clinics, owing to different forms of cognitive problems. Some patients were referred from other memory clinics in Sweden to seek a second opinion. Most patients referred by GPs had undergone cognitive tests as Mini-Mental State Examination (MMSE), structural imaging (computed tomography, CT), and blood analysis, while patients referred from other clinics had usually undergone less thorough assessments. At their first visit, patients underwent physical, neurological, psychiatric, and cognitive assessments, and a detailed medical history was recorded. Patients underwent diagnostic workup for a tertiary memory clinic, which includes neuropsychological testing, magnetic resonance imaging (MRI), and CSF sampling with analysis of the major CSF biomarkers for AD pathology and in a subset of cases also apolipoprotein E (APOE) genotyping. Amyloid PET imaging with flutemetamol was performed in cases where the diagnosis still remained uncertain after FDG-PET.

A baseline clinical diagnosis was made in consensus following memory assessment by a dementia expert team. The main reasons for performing FDG-PET were an atypical clinical presentation, uninformative/contradictory biomarkers’ results, or absence of CSF analysis. After disclosure of the FDG-PET result, a consensus meeting between specialists in cognitive disorders, clinical neuropsychologists, and specialist nurses revised the diagnosis, taking into account also all the available information from the original memory assessment, all other biomarkers, neuropsychological testing, and additional clinical information collected during the time interval between the baseline diagnosis and FDG-PET scan. The nuclear medicine physician was not present at the diagnostic consensus meeting but reported to the referring clinician the FDG-PET patterns (with suggestion of diagnosis in probable cases) and both visual and semiquantitative images. Patients might fulfill criteria for dementia or not [10]. Diagnostic categories for non-demented patients included memory syndrome (MS), if the patient did not complete the routine diagnostic work up, or MCI [11]. Diagnostic categories for dementia included typical AD [1, 12], logopenic variant of primary progressive aphasia (lvPPA) [4, 12], posterior cortical atrophy (PCA) [12], behavioral variant of frontotemporal dementia (bvFTD) [3], semantic variant of primary progressive aphasia (svPPA) [4], non-fluent variant of primary progressive aphasia (nfPPA) [4], dementia with Lewy bodies (DLB) [2], Parkinson disease dementia (PDD) [2], corticobasal degeneration (CBD) [5], progressive supranuclear palsy (PSP) [13], parkinsonian syndrome (PS)—if no specific parkinsonian disorders could be diagnosed, amyotrophic lateral sclerosis with cognitive impairment (ALSci) [14], spinocerebellar ataxia (SCA), vascular dementia (VD) [15], and dementia of unclear etiology (not otherwise specified), also known as “dementia NOS”.

### Baseline diagnostic assessment

Most patients completed a large battery of neuropsychological tests covering different cognitive domains [16]. These included the MMSE as well as components of the Wechsler Adult Intelligence Scale, Revised (WAIS-R; information and similarities, logical memory, block design and digit symbol), figure classification, subtest of the Synonyms Reasoning Block Test (SRB2), Rey Auditory Verbal Learning Test (RAVLT), copying and memory subtests of the Rey-Osterrieth Complex Figure Test (ROCFT), parts A and B of the Trail Making Test (TMT), and/or the Verbal Fluency Test (FAS).

CT or MR imaging was performed at various radiology departments in Stockholm using different platforms and protocols. Cerebral atrophy was assessed clinically by experienced neuroradiologists at the Department of Radiology, Karolinska University Hospital, according to standard visual rating scales. Atrophy of the medial temporal lobe was evaluated using the medial temporal atrophy (MTA) scale [17]. Overall cortical atrophy was assessed using the global cortical atrophy (GCA) scale [18].

CSF samples were collected via lumbar puncture in 237 out of 277 patients (85.5%) (53% and 89% of patients diagnosed with MCI and dementia at baseline, respectively) under non-fasting conditions as part of routine memory assessment. The lack of CSF biomarkers in 40 patients was due to the use of anticoagulants, spinal stenosis or related problems, refusal to undergo CSF sampling, or technical problems. The CSF samples were routinely analyzed at the Clinical Neurochemistry Laboratory, Sahlgrenska University Hospital, Mölndal, Sweden, for Aβ1–42, t-tau, and p-tau, using commercially available ELISAs (INNOTEST; Fujirebio, Ghent, Belgium). Internal cut-off values of 550 ng/L for Aβ1–42, 400 ng/L for t-tau, and 80 ng/L for p-tau were used.

### FDG-PET acquisition and analysis

FDG-PET was performed after 4.7 ± 6.0 (mean ± SD) months from the baseline diagnosis (4.2 ± 4.3 months and 5.5 ± 8.4 months in patients diagnosed with MCI and dementia at baseline, respectively). FDG-PET investigations were performed at the Department of Medical Radiation Physics and Nuclear Medicine Imaging, Karolinska University Hospital, Stockholm, Sweden, using a Biograph mCT PET/CT scanner (Siemens/CTI, Knoxville, TN). FDG-PET was performed with patient with open eyes, as a 10-min or 15-min list-mode scan starting 30 to 45 min after intravenous injection of 2–3 MBq/kg. All appropriate corrections including time-of-flight were applied, with a low-dose CT scan used for attenuation correction. Images were reconstructed using ordered subset expectation maximization (OSEM; five iterations, 21 subsets, 2.0 mm Gaussian filter). According to clinical routine assessment, summation images were visually analyzed, supported by a semiquantitative analysis in the form of standardized uptake value ratios (SUVR) based on automatically generated regions of interest (cortical and subcortical regions, normalized to whole brain) and voxel-wise Z-score stereotactic surface projection analysis (Siemens Syngo.via software). Brain metabolic patterns were classified by a board-certified nuclear medicine physician according to the previous literature. The rater had to make a forced decision among different patterns, based on the topography of hypometabolism described in the clinical/research criteria of each dementing condition: possible/probable AD (this category included “typical AD” pattern [1], “lv-PPA” pattern [4], and “PCA” pattern [12]); possible/probable FTLD (this category included “bvFTD” pattern [3], “svPPA” pattern [4], “nfPPA” pattern [4], “CBD” pattern [5], and “PSP” pattern [13]); possible/probable DLB [2]; when FDG-PET scans were not suggestive of any of the patterns described above, the rater classified the scans as either negative for neurodegenerative disease; slight hypometabolic abnormalities not suggestive for a specific disease; or widespread hypometabolism not typical for any specific disease. Based on the degree of confidence of the nuclear medicine physician, metabolic patterns deemed as frankly indicative of AD, FTLD, or DLB were considered as probable for the respective disease whereas less obvious metabolic changes but still suspicious for a specific type of neurodegeneration were considered as possible for AD, FTLD, or DLB.

### Flutemetamol PET

Flutemetamol PET was performed as part of the clinical assessment in 52 out of the 277 patients (18.8%) (23% and 11% of patients diagnosed with MCI and dementia at baseline, respectively). These studies were performed after FDG-PET in 47 patients (90%) and prior to FDG-PET in 5 patients. Flutemetamol scans were performed using the same scanner as that used for FDG-PET. Flutemetamol PET was performed as a 20-min list-mode scan 90 min after injection of 185 MBq flutemetamol. Data were corrected and images reconstructed in an identical way as the in the FDG-PET studies. Flutemetamol summation images were visually assessed as positive (abnormal) or negative (normal) by the same nuclear medicine physician.

### Outcome variables and statistical analysis

Outcome variables of this study were the clinical diagnoses, assessed by dementia experts 3 times throughout the study. The first time corresponded to the baseline clinical diagnosis. At time 2, physicians had received the FDG-PET result and subsequently reformulated the diagnosis, considering FDG-PET result as well as all other biomarkers, neuropsychological testing and additional clinical information obtained during the time interval between the baseline diagnosis and FDG-PET scan. Time 3 referred to the last diagnosis available at the follow-up (reference diagnosis). Since patients diagnosed with MS and MCI did not differ for demographic, clinical, and biomarkers measures at baseline and presented similar percentage change in diagnosis post- compared to pre-FDG-PET, they were pooled together in the “MCI” group. All clinical variants of AD (typical AD, lvPPA, PCA) were pooled in the “AD” group; bvFTD, svPPA, nfPPA, CBD, PSP, and ALSci were pooled in the “FTLD” group; DLB and PDD were pooled together in the “DLB” group. All the remaining diagnoses were pooled in the “other dementia” group. Clinical impact of FDG-PET was evaluated in terms of percentage change in diagnosis (baseline vs. post-FDG-PET). In addition, and for the group of patients diagnosed with dementia at baseline only, incremental diagnostic value of FDG-PET was evaluated in terms of change in diagnostic accuracy when comparing each baseline and post-FDG-PET diagnoses to a long-term follow-up diagnosis used as reference. The prognostic and diagnostic performance testing of FDG-PET and other biomarkers were also assessed through measures dependent and independent on the prevalence of the disease (i.e., sensitivity, specificity, accuracy vs. likelihood ratios, respectively).

Demographic and clinical characteristics were compared between diagnostic groups using ANOVA and the Wilcoxon signed ranks test for continuous variables. Differences in categorical variables were assessed using Fisher’s exact test. Bonferroni correction was used to control for multiple comparisons in post hoc analyses. Change in diagnostic accuracy was assessed using the McNemar test. Statistical computations were performed using R v. 3.5.3 (The R Foundation for Statistical Computing), with two-sided *p* values <0.05 considered to indicate significance.

## Results

### Patient characteristics at baseline

Demographic, clinical, and biomarker measures at baseline in the 277 patients are presented in Table 1. At baseline, 64% of patients were diagnosed with MCI (mean age, 64.9 years; 55% female) and 36% with dementia (mean age, 67.4 years; 46% female). Mean scores on MMSE were 26.2 out of 30 in MCI and 22.2 out of 30 in dementia cases. In the dementia group, 34% of patients was diagnosed with AD, 32% with dementia NOS, 14% with FTLD, 10% with DLB, and 10% with other dementia. The whole patient group was considered with uncertain diagnosis, and mean CSF Aβ1–42 values were in the normal range in all groups. CSF p-tau and t-tau values as well as atrophy scale scores were also in similar range among the groups, after post hoc correction for multiple comparisons.

**Table 1:** Demographic, clinical and biomarkers measures at baseline (before FDG-PET), grouped by baseline diagnosis

	MCI diagnosis at baseline	Uncertain dementia diagnoses at baseline				
	MCI	AD	FTLD	DLB	Dem NOS	Other
	(n = 177)	(*n* = 34)	(*n* = 14)	(n = 10)	(*n* = 32)	(n = 10)
Age^a^, mean (sd), years	64.9 (9.8)	67.6 (8.3)	64.6 (7.2)	73.7 (7.6)	65.8 (10.1)	68.7 (7.9)
Gender, *N*. (%)						
Female	98 (55)	17 (50)	7 (50)	3 (30)	14 (44)	5 (50)
Male	79 (45)	17 (50)	7 (50)	7 (70)	18 (56)	5 (50)
Follow-up, mean (sd), y	3.5 (1.8)	4.1 (1.9)	4.3 (1.4)	3.5 (2.0)	3.5 (1.8)	3.7 (1.8)
MMSE^b^, mean (sd)	26.2 (3.5)	22.1 (6.4)	24.3 (5.5)	22.1 (6.8)	21.1 (6.1)	24.5 (5.3)
APOE, *N*. (%)						
*N* subjects	118	24	5	4	22	5
Carriers E4	61 (52)	17 (71)	3 (60)	3 (75)	9 (41)	1 (20)
Non-Carriers E4	57 (48)	7 (29)	2 (40)	1 (25)	13 (59)	4 (80)
MTA, *N*. (%)						
*N* subjects	158	30	11	7	29	8
0	22 (14)	0	0	0	1 (3)	1 (13)
1	69 (44)	10 (33)	4 (36)	1 (14)	9 (31)	6 (75)
2	41 (26)	14 (47)	6 (55)	4 (57)	11 (38)	1 (13)
3	21 (13)	5 (17)	1 (9)	2 (29)	5 (17)	0
4	5 (3)	1 (3)	0	0	3 (10)	0
GCA, *N*. (%)						
*N* subjects	136	23	11	6	26	8
0	12 (9)	0	0	0	1 (4)	0
1	63 (46)	12 (52)	3 (27)	2 (33)	8 (31)	4 (50)
2	54 (40)	8 (35)	6 (55)	4 (67)	14 (54)	4 (50)
3	7 (5)	3 (13)	2 (18)	0	3 (12)	0
CSF biomarkers, mean (sd), ng/L						
*N* subjects	148	31	12	8	29	9
Aβ1–42^c^	781.1 (306.7)	710.7 (349.5)	1034.8 (274.0)	936.2 (458.3)	922.6 (350.8)	976.2 (306.8)
p-tau	65.1 (49.4)	68.9 (39.9)	55.2 (26.1)	47.6 (16.7)	55.2 (20.8)	41.5 (23.8)
t-tau	428.3 (276.9)	508.7 (358.3)	403.0 (223.2)	306.4 (120.4)	385.4 (186.3)	258.9 (124.4)

*AD* Alzheimer’s disease, *Dem NOS* dementia not otherwise specified, *DLB* dementia with Lewy bodies, *FTLD* frontotemporal lobar degeneration, *GCA* global cortical atrophy, *MMSE* Mini-Mental State Examination, *MTA* medial temporal atrophy

ANOVA/Wilcoxon tests and Fisher tests (with Bonferroni post hoc correction) (excluding “Other” group)

^a^Age: DLB > MCI (*p* < 0.05)

^b^MMSE: MCI > AD and Dem NOS (*p* < 0.001)

^c^Aβ1–42: MCI and AD < FTLD (*p* < 0.05)

### Clinical impact of FDG-PET

**Fig. 1:**
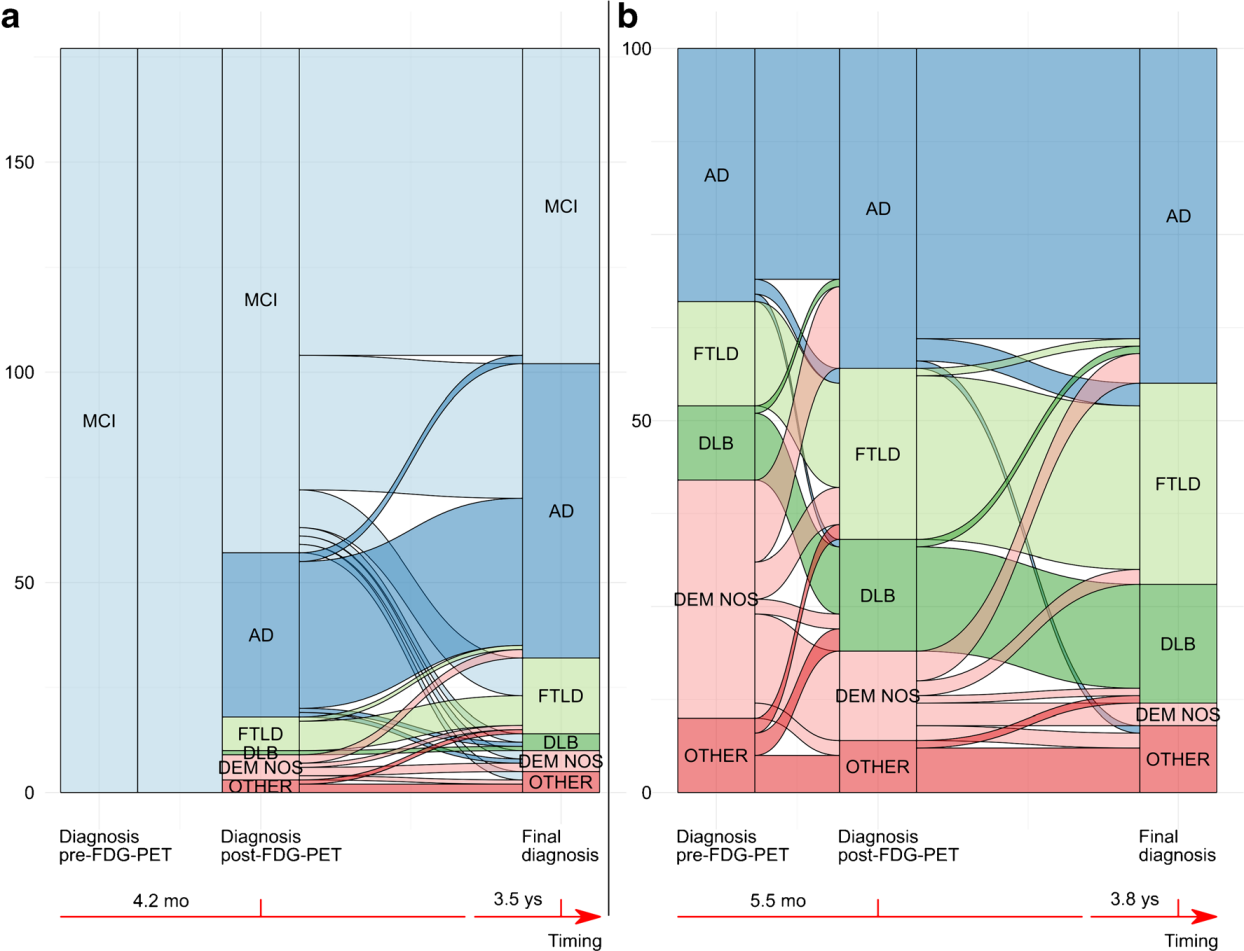
Alluvial diagrams illustrating the change in diagnosis. *AD*, Alzheimer’s disease; *Dem NOS*, dementia not otherwise specified; *DLB*, dementia with Lewy bodies; *FTLD*, frontotemporal lobar degeneration; *MCI*, mild cognitive impairment. Changes in diagnosis from pre- to post-FDG-PET imaging, and to the final diagnosis after 3.6 years follow-up time in patients diagnosed with MCI (*n* = 177) (**a**) and uncertain dementia diagnoses (*n* = 100) (**b**) at baseline.

In the whole selected population of memory clinic patients with an uncertain diagnosis at baseline, FDG-PET disclosure resulted in a change of diagnosis in 86 of 277 (31%) cases (Fig. 1, Table 2). The diagnosis of MCI was changed in 57 out of 177 subjects (32%). The diagnosis changed mostly toward AD (39/57, 68%), followed by FTLD (8/57, 14%), dementia NOS (6/57, 11%), other dementia (3/57, 5%), and DLB (1/57, 2%). Interestingly, even an FDG-PET pattern characterized by slight abnormalities led to a change from a diagnosis of MCI to one of dementia in 11 out of 57 (19%) MCI cases. In patients with uncertain dementia diagnosis at baseline, the diagnosis changed in 29 out of 100 (29%) patients. Of note, post-FDG-PET diagnostic change was greater than twofold in the sub-sample of cases with Dem NOS (20/32, 63%) (11 AD, 5 FTLD, 2 DLB, and 2 other dementia), while less frequently in other dementia (5/10, 50%) (2 FTLD, 3 DLB), AD (in 3/34, 9%) (2 FTLD and 1 DLB), DLB (1/10, 10%) (1 AD), and in none of FTLD (0/14) patients.

**Table 2:** Clinical impact of FDG-PET in terms of changes in diagnosis from pre- to post-FDG-PET imaging, according to FDG-PET classification

Diagnosis pre-FDG-PET	Change in diagnosis post- vs pre-FDG-PET	FDG-PET classification	Number of those with change	Diagnosis post-FDG-PET	Final diagnosis (at 3.6-year follow-up)
MCI (*n* = 177)	57/177 (32%)	Slight abnormalities	11	8 AD, 1 FTLD, 1 Dem NOS, 1 Other	7 AD, 1 FTLD, 2 Dem NOS, 1 Other
		Possible AD	10	9 AD, 1 Other	7 AD, 1 FTLD, 2 MCI
		Probable AD	18	16 AD, 2 Dem NOS	16 AD, 1 FTLD, 1 DLB
		Possible FTLD	8	4 FTLD, 1 AD, 3 Dem NOS	4 FTLD, 2 AD, 1 Dem NOS, 1 Other
		Probable FTLD	2	2 FTLD	2 FTLD
		Probable DLB	1	1 DLB	1 DLB
		Widespread hypometabolism	7	5 AD, 1 FTLD, 1 Other	6 AD, 1 Other
AD (*n* = 34)	3/34 (9%)	Probable AD	2	1 FTLD, 1 DLB	1 FTLD, 1 DLB
		Possible FTLD	1	1 FTLD	1 FTLD
FTLD (*n* = 14)	0/14 (0%)				
DLB (*n* = 10)	1/10 (10%)	Probable AD	1	1 AD	1 AD
Dem NOS (*n* = 32)	20/32 (63%)	Negative	1	1 Other	1 Other
		Slight abnormalities	1	1 AD	1 AD
		Possible AD	1	1 AD	1 AD
		Probable AD	7	7 AD	7 AD
		Possible FTLD	2	2 AD	1 FTLD, 1 AD
		Probable FTLD	5	5 FTLD	5 FTLD
		Possible DLB	1	1 DLB	1 AD
		Probable DLB	1	1 DLB	1 DLB
		Widespread hypometabolism	1	1 Other	1 Other
Other (*n* = 10)	5/10 (50%)	Probable FTLD	1	1 FTLD	1 FTLD
		Possible FTLD	2	1 FTLD, 1 DLB	1 FTLD, 1 DLB
		Possible DLB	1	1 DLB	1 DLB
		Probable DLB	1	1 DLB	1 DLB

*AD* Alzheimer’s disease, *Dem NOS* dementia not otherwise specified, *DLB* dementia with Lewy bodies, *FTLD* frontotemporal lobar degeneration

In subjects diagnosed with MCI at baseline, when the diagnosis changed after FDG-PET, both probable and possible AD-like patterns led to a change toward an AD diagnosis in the majority of cases (16/18 and 9/10, respectively). In 2/2 MCI subjects with probable FTLD-like pattern, the diagnosis changed to FTLD, whereas a possible FTLD-like pattern led to a FTLD diagnosis only in half of cases (4/8). Similarly, in patients with baseline diagnosis of dementia NOS, an AD-like pattern led to an AD diagnosis in all patients, while a FTLD-like pattern changed diagnosis toward FTLD only in probable cases.

The disclosure of FDG-PET led not only to a change in diagnosis but also to more patients receiving treatment with cholinesterase inhibitors (ChEIs) and memantine. In the whole population of memory clinic patients at baseline (*n* = 277, including all MCI and uncertain dementia diagnosis), a total of 19 patients (4 MCI, 9 AD, 2 DLB, and 4 Dem NOS) were receiving treatment with ChEIs/memantine prior to FDG-PET examination. After FDG-PET imaging and revision of initial diagnosis, 57 patients out of the total 277 investigated in this study were receiving treatment with ChEIs/memantine. Therefore, FDG-PET imaging resulted in a threefold increase in number of patients receiving treatment when considering the whole population. From the group of patients diagnosed with MCI at baseline (*n* = 177), only 4 were receiving medication prior to FDG-PET; after FDG-PET and revision of initial diagnosis, 29 patients (6 MCI, 19 AD, 1 FTLD, 1 DLB, and 2 Dem NOS) were receiving treatment. From the group of patients with uncertain dementia diagnosis at baseline (*n* = 100), 15 were receiving medication prior to FDG-PET; after FDG-PET and revision of initial diagnosis, 28 patients (18 AD, 1 FTLD, 4 DLB, and 5 Dem NOS) were receiving treatment.

### Patient characteristics at follow-up

Mean follow-up time was similar for MCI and dementia cases (3.5 ± 1.8 and 3.8 ± 1.8 years, respectively). At follow-up, 102 out of 177 MCI subjects (58%) had developed dementia, namely 70/177 had converted to AD, 18/177 to FTLD, 4/177 to DLB, 5/177 to dementia NOS, and 5/177 to other dementia (Online Resource 1). Among subjects diagnosed with MCI at baseline, only the patients who converted to AD at follow-up had shown abnormal mean CSF p-tau and t-tau values at baseline, while in the other diagnostic groups, all mean CSF biomarker values at baseline were normal. Atrophy scale scores were similar among groups, after post hoc correction for multiple comparisons. In the subgroup of patients who underwent flutemetamol PET, amyloid positivity was observed in 21 out of 21 (100%) patients who converted to AD and 5 out of 14 (36%) stable MCI, while in 0 out of 6 (0%) patients who converted to FTLD, DLB, dementia NOS, and other dementia.

Among patients diagnosed with dementia at baseline, mean CSF Aβ1–42 and p-tau values were all in the normal range in all groups due to the high prevalence of uninformative/contradictory CSF analysis in our selected study population, while mean CSF t-tau values were abnormal in cases finally diagnosed with AD (Online Resource 2). Atrophy scales were similar among the different diagnostic groups. Flutemetamol PET was positive in 5 out of 6 patients finally diagnosed with AD, in 1 out of 3 patients finally diagnosed with FTLD and in none of the cases finally diagnosed with “other dementia.”

### Incremental diagnostic value of FDG-PET

Among patients diagnosed with dementia at baseline, the accuracy of the clinical diagnosis with respect to the final diagnosis at follow-up increased from the pre- to the post-FDG-PET diagnosis: from 77 to 90% in patients with AD and from 85 to 94% in patients with FTLD (*p* < 0.01, McNemar test) and improved with a trend toward significance in patients with DLB (Table 3).

**Table 3:** Incremental diagnostic value of FDG-PET, in terms of comparing each of the pre- and post-FDG-PET accuracy to the final follow-up diagnosis as the reference, in the group of patients with uncertain dementia diagnoses at baseline (*n* = 100)

	Diagnosis AD vs non-AD		Diagnosis FTLD vs non-FTLD		Diagnosis DLB vs non-DLB	
	Pre-FDG-PET	Post-FDG-PET	Pre-FDG-PET	Post-FDG-PET	Pre-FDG-PET	Post-FDG-PET
SE (%)	62	87	48	82	56	88
SP (%)	89	93	99	99	99	99
ACC (%)^a^	77	90	85	94	92	97
LR+	5.7	11.9	34.3	58.2	46.8	72.9
LR−	0.4	0.1	0.5	0.2	0.4	0.1

*ACC* accuracy, *AD* Alzheimer’s disease, *DLB* dementia with Lewy bodies, *FTLD* frontotemporal lobar degeneration, *LR* likelihood-ratio, *SE* sensitivity, *SP* specificity

LR+ > 5 indicates that the biomarker positive classification is associated with the disease occurrence. LR− < 0.2 indicates that the biomarker negative classification is associated with the absence of the disease

^a^McNemar’s test

Accuracy of AD vs non AD diagnosis improved after FDG-PET (*p* < 0.01)

Accuracy of FTLD vs non FTLD diagnosis improved after FDG-PET (*p* < 0.01)

Accuracy of DLB vs non DLB diagnosis did not significantly improve after FDG-PET

### FDG-PET classification

FDG-PET patterns were assessed as negative for neurodegenerative disease only in 8 out of 277 patients (3%), including 7 patients diagnosed with MCI at baseline (follow-up diagnosis: stable MCI in 6 cases, converter to other dementia in 1 case) and 1 patient diagnosed with dementia at baseline (as other dementia at follow-up). For both MCI and dementia groups at baseline, a significant association was found between the AD, FTLD, and DLB FDG-PET patterns assessment and a follow-up diagnosis of AD, FTLD, and DLB, respectively (Online Resource 1 and Online Resource 2). The majority (49%) of stable MCI showed an FDG-PET pattern characterized by slight abnormalities, while they less frequently showed FTLD patterns (25%) (15 possible and 4 probable patterns), AD patterns (15%) (10 possible and 1 probable patterns), negative for neurodegeneration pattern (8%), and widespread hypometabolism pattern (3%).

Considering that a positive likelihood ratio (LR+) >5 indicates that the biomarker positive classification is associated with the disease occurrence, FDG-PET performed better and had a relevant association with the presence of the dementia condition at follow-up in dementia patients compared to MCI subjects (LR+ <5 and > 5, respectively) (Table 4). When comparing the accuracy of FDG-PET versus CSF biomarkers in regard to their accuracy to detect AD converters, FDG-PET showed higher accuracy than CSF Aβ1–42, t-tau, and p-tau biomarkers individually. On the contrary, FDG-PET showed lower accuracy than flutemetamol PET (Table 5).

**Table 4:** FDG-PET classification accuracy in detecting AD and FTLD converters, in the group of patients with MCI at baseline (*n* = 177) and in detecting AD, FTLD, and DLB, in the group of patients with uncertain dementia diagnoses at baseline (*n* = 100)

	FDG-PET in MCI subjects at baseline (*n* = 177)		FDG-PET in subjects with uncertain dementia diagnoses at baseline (*n* = 100)		
	AD vs non-AD	FTLD vs non-FTLD	AD vs non-AD	FTLD vs non-FTLD	DLB vs non-DLB
SE (%)	61	78	76	82	75
SP (%)	85	84	95	90	95
ACC (%)	76	83	86	88	92
LR+	4.1	4.7	13.7	8.5	15.6
LR−	0.4	0.3	0.2	0.2	0.3

*ACC* accuracy, *AD* Alzheimer’s disease, *DLB* dementia with Lewy bodies, *FTLD* frontotemporal lobar degeneration, *LR* likelihood ratio, *SE* sensitivity, *SP* specificity

**Table 5:** Biomarkers accuracy in detecting AD converters, in the group of patients with MCI at baseline (*n* = 177)

	AD converters vs non-AD converters				
	FDG-PET	Aβ1–42	p-tau	t-tau	Flutemetamol PET
SE (%)	61	43	46	75	100
SP (%)	85	89	86	74	75
ACC (%)	76	70	69	74	88
LR+	4.1	4.0	3.3	2.9	4.0
LR−	0.4	0.6	0.6	0.3	0

*ACC* accuracy, *AD* Alzheimer’s disease, *LR* likelihood ratio, *SE* sensitivity, *SP* specificity

## Discussion

In this study, we assessed the utility of FDG-PET imaging in a selected clinical population of a tertiary memory clinic, by investigating a large population of cognitively impaired patients with uncertain diagnosis who met appropriate use criteria for FDG-PET. Our study aimed to provide real-world evidence in this clinical setting on the utility of FDG-PET, assessed over a comprehensive baseline diagnosis including neuropsychological testing, MRI and CSF biomarkers, according to the routine praxis at the clinic. Firstly, we found that FDG-PET led to a significant change in diagnosis in 31% of the whole patient population, with the strongest impact on patients with a diagnosis of dementia “of unclear etiology” (63%) and MCI subjects (32%). Secondly, we found that FDG-PET provides significant incremental diagnostic value, improving the diagnostic accuracy with respect to an extended follow-up diagnosis used as reference, as recently recommended [9]. The incremental value of FDG-PET was confirmed both when considering AD and FTLD dementias as outcome measures. Finally, we showed that FDG-PET disease-specific hypometabolism patterns perform better in dementia than in MCI, in predicting the final clinical diagnosis at follow-up (positive likelihood ratios >5 and < 5, respectively).

The present study focused on a unique cohort of patients in whom, despite extensive assessment including CSF Aβ1–42, p-tau, and t-tau biomarkers, the diagnosis remained uncertain. The fact that our cohort does not represent a research population but a real-world clinical population (that met appropriate use criteria for FDG-PET) represents a strength of this study. Previously, one study had investigated the change in clinical diagnosis from pre- to post-FDG-PET in a smaller cohort of 94 patients with a baseline diagnosis of MCI, atypical/unclear dementia, or typical dementia [19]. In that study, the visual assessment of FDG-PET was not always supported by a semi-quantitative analysis. Also, the baseline diagnosis of the previous report was based on neurological and neuropsychological examinations supported by MRI alone, without CSF biomarkers. The differences in study designs may explain the higher rate of diagnostic change after FDG-PET in MCI subjects in our study (32%) compared to the previous report (8%) [19]. Notably, among MCI subjects in our study, even an FDG-PET visual assessment of slight abnormalities led to a change to a dementia diagnosis in 19% of cases. Therefore, diagnostic change in our MCI subjects was likely facilitated by the integration of FDG-PET information with available pathophysiological biomarkers. Consistent with that previous report [19], we found low occurrence of diagnostic change in cases with a preliminary diagnosis of specific dementia, while the largest diagnostic change was in cases with dementia “of unclear etiology” (63%) and in atypical/unclear dementia (60%).

Previous studies have compared the baseline diagnosis and FDG-PET classification to the final diagnosis provided by either a follow-up investigation or by the neuropathological diagnosis [20–23]. Nevertheless, we have not found any study investigating the change in diagnostic accuracy when comparing pre- and post-FDG-PET diagnosis to a common reference standard, which is considered critical to evaluate the incremental diagnostic value [9]. Moreover, previous reports are limited and strictly focused on conversion to AD dementia and on FDG-PET incremental value in the diagnosis of AD vs. non-AD, often due to small sample sizes of non-AD cases. In this regard, we report for the first time that among demented patients with uncertain diagnosis, after results of FDG-PET, diagnostic accuracy significantly improved from 77 to 90% in AD and from 85 to 94% in FTLD patients (*p* < 0.01). In DLB patients, diagnostic accuracy improved with a trend toward significance (from 92 to 97%). Previously, one study found that FDG-PET accuracy was superior to a baseline clinical evaluation of AD and similar to the final follow-up evaluation [20]. Two other studies found that amyloid PET contributed more to diagnostic changes than FDG-PET [21, 22]. Finally, one study found that amyloid PET provided significant incremental diagnostic value beyond clinical and FDG-PET diagnoses of AD [23]. In our study, in the subgroup of patients who underwent amyloid PET, the flutemetamol PET was performed mostly after the post-FDG-PET diagnosis, except in 11 out of 52 cases (and among these 11 cases, 6 had their diagnosis changed after FDG-PET). Given the high clinical impact of amyloid PET [24], for these 6 cases alone, we cannot distinguish the FDG-PET from the flutemetamol PET incremental diagnostic value. Analyses excluding the 11 cases are almost unchanged (Online Resource 3). Of note, with our study, we elucidated the FDG-PET incremental value also in non-AD conditions, where the lack of pathophysiological biomarkers strengthen the need of assessing the role of FDG-PET. Recent research has shown that amyloid PET tracers could have dual utility, where the early-phase uptake (first few minutes of the PET scan) could provide a surrogate for brain perfusion or metabolism [25]; however, more studies are needed to assess the potential contribution of this approach to increase the clinical utility of amyloid PET.

Performance values of FDG-PET for both early and differential diagnosis of dementia are strongly dependent on the analytical method used, with improved performance values reported when semi-quantitative approaches are used [26]. In our study, we provide evidence on the performance of FDG-PET at different stages of dementia in a large cohort, using visual assessment supported by a semi-quantitative analysis according to routine clinical praxis. Considering that a positive biomarker classification is associated with the disease occurrence when LR+ >5, we found that in dementia patients there was a strong association between FDG-PET pattern and AD, FTLD, and DLB occurrence (LR+ 13.7, 8.5, and 15.6, respectively). In MCI subjects, FDG-PET showed a weaker association in detecting prodromal AD and FTLD (LR+ 4.1 and 4.7, respectively). In this regard, a recent review identified a substantial variability in detecting MCI due to AD [8] (SE 38–98%, SP 41–97%, ACC 58–100%, with only one paper reporting LR values of LR+ 8.14 and LR− 0.12) [27–39]. Moreover, very limited evidence is available in detecting MCI due to non-AD conditions, often due to a small sample size [8]. Semi-automatic assessment of FDG-PET in one study correctly identified at baseline the only two MCI subjects that later converted to FTLD [40] and in another study it correctly identified at baseline 7 out of 10 (70%) MCI subjects that later converted to FTLD [41], similar to our findings (14 out of 18 (78%)).

When comparing the performance of FDG-PET versus other CSF and PET biomarkers in regard to their accuracy to detect AD converters, FDG-PET performed better than CSF Aβ1–42 (LR+ 4.0), p-tau (LR+ 3.3), and t-tau (LR+ 2.9) biomarkers individually, but worse than flutemetamol PET (LR+ 4.0). Although our study, by design, was predisposed to uninformative/contradictory CSF analysis, our results align well with previous findings showing FDG-PET to be more accurate in detecting AD converters than CSF Aβ1–42, p-tau, and t-tau [33, 41–43]. Crucially, and consistent with recent findings, all biomarkers of prediction to AD conversion showed individually LR+ <5, which means that they all confer only a slight increase in the probability to detect AD in the case of a positive test [41]. The combined use of FDG-PET and CSF biomarkers [33, 34, 41], but also FDG-PET and amyloid PET information [44], led to an increase in their diagnostic effectiveness, likely because the different biomarkers provide complementary information.

This study has some limitations. First, as our study is retrospective, we could not assess changes in diagnostic confidence in addition to changes in clinical diagnosis, assuming an increased diagnostic confidence as a prerequisite for changing a diagnosis. This outcome has been recently considered in evaluating the incremental diagnostic value of amyloid PET [45, 46]. Assessing diagnostic confidence would be important in particular to evaluate the utility of FDG-PET in the diagnosis of prodromal stages of dementia [47]. Then, as a clinical study, the time interval between the baseline diagnosis and FDG-PET scan was variable and sometimes long enough that changes in the clinical picture cannot be ruled out. In addition, since both post-FDG-PET diagnosis and final diagnosis incorporated FDG-PET results, the estimated accuracy between the two might be slightly overestimated. Still, that would be minimal, as our gold standard, i.e., the final clinical diagnosis, was based on various information aside from FDG-PET, including CSF biomarkers as well as amyloid PET, cognitive tests, and repeated clinical assessments at a long follow-up. Finally, we acknowledge that FDG-PET and flutemetamol PET scans have been evaluated by the same single board-certified nuclear medicine physician working routinely with diagnostics of neurodegenerative disease. However, the use of a visual assessment supported by a semi-quantitative analysis strengthens the translatability of our results, and since evaluation of FDG-PET and flutemetamol PET scans is based on independent and modality-specific criteria, we believe this has not introduced any systematic bias in the current study.

## Conclusion

Worldwide, a great effort is ongoing to estimate and understand the clinical utility of biomarkers in the workflow of cognitive disorders, with the greatest advances in the field of AD. With this study, we have provided real-world evidence that within a selected clinical population of cognitively impaired patients with an uncertain diagnosis, FDG-PET made a significant contribution to improve early and differential diagnosis of uncertain dementia, with the strongest impact being among patients with a diagnosis of dementia “of unclear etiology”. Notwithstanding the relatively lower performance of FDG-PET in the MCI compared to the dementia condition, FDG-PET contributed to change the diagnosis from MCI to prodromal forms of dementia, when added to a comprehensive assessment. When overt dementia is present, we have shown that FDG-PET had an added value in the diagnosis of AD, in cases showing absence or uninformative/contradictory CSF analysis. Diagnostic utility of FDG-PET in FTLD is of particular significance, also considering the paucity of pathophysiological biomarkers in this disease.

## Electronic supplementary material

ESM 1 (DOCX 19 kb)

ESM 2 (DOCX 18 kb)

ESM 3 (DOCX 23 kb)

## Data Availability

Anonymized data will be shared by request from any qualified investigator for the sole purpose of replicating procedures and results presented in the report provided that data transfer is in agreement with EU legislation on the general data protection regulation.

## References

[1] McKhann G, Knopman D, Chertkow H, Hyman B, Jack CJ, Kawas C (2011). The diagnosis of dementia due to Alzheimer’s disease: recommendations from the National Institute on Aging-Alzheimer’s Association workgroups on diagnostic guidelines for Alzheimer’s disease. Alzheimers Dement.

[2] McKeith I, Boeve B, Dickson D, Halliday G, Taylor J, Weintraub D (2017). Diagnosis and management of dementia with Lewy bodies: fourth consensus report of the DLB Consortium. Neurology..

[3] Rascovsky K, Hodges JR, Knopman D, Mendez MF, Kramer JH, Neuhaus J (2011). Sensitivity of revised diagnostic criteria for the behavioural variant of frontotemporal dementia. Brain..

[4] Gorno-Tempini ML, Hillis AE, Weintraub S, Kertesz A, Mendez M, Cappa SF (2011). Classification of primary progressive aphasia and its variants. Neurology..

[5] Armstrong MJ, Litvan I, Lang AE, Bak TH, Bhatia KP, Borroni B (2013). Criteria for the diagnosis of corticobasal degeneration. Neurology..

[6] Nestor PJ, Altomare D, Festari C, Drzezga A, Rivolta J, Walker Z (2018). Clinical utility of FDG-PET for the differential diagnosis among the main forms of dementia. Eur J Nucl Med Mol Imaging.

[7] Nobili F, Arbizu J, Bouwman F, Drzezga A, Agosta F, Nestor P (2018). European Association of Nuclear Medicine and European Academy of neurology recommendations for the use of brain 18 F-fluorodeoxyglucose positron emission tomography in neurodegenerative cognitive impairment and dementia: Delphi consensus. Eur J Neurol.

[8] Arbizu J, Festari C, Altomare D, Walker Z, Bouwman F, Rivolta J (2018). Clinical utility of FDG-PET for the clinical diagnosis in MCI. Eur J Nucl Med Mol Imaging.

[9] Boccardi M, Festari C, Altomare D, Gandolfo F, Orini S, Nobili F (2018). Assessing FDG-PET diagnostic accuracy studies to develop recommendations for clinical use in dementia. Eur J Nucl Med Mol Imaging.

[10] American Psychiatric Association (2013). Diagnostic and Statistical Manual of Mental Disorders (DSM-5).

[11] Winblad B, Palmer K, Kivipelto M, Jelic V, Fratiglioni L, Wahlund L (2004). Mild cognitive impairment--beyond controversies, towards a consensus: report of the International Working Group on Mild Cognitive Impairment. J Intern Med.

[12] Dubois B, Feldman HH, Jacova C, Hampel H, Molinuevo JL, Blennow K (2014). Advancing research diagnostic criteria for Alzheimer’s disease: the IWG-2 criteria. Lancet Neurol.

[13] Hoglinger GU, Respondek G, Stamelou M, Kurz C, Josephs KA, Lang AE (2017). Clinical diagnosis of progressive supranuclear palsy: the movement disorder society criteria. Mov Disord.

[14] Strong MJ, Abrahams S, Goldstein LH, Woolley S, Mclaughlin P, Snowden J (2017). Amyotrophic lateral sclerosis - frontotemporal spectrum disorder (ALS-FTSD): revised diagnostic criteria. Amyotroph Lateral Scler Front Degener.

[15] Román G, Tatemichi T, Erkinjuntti T, Cummings J, Masdeu J, Garcia J (1993). Vascular dementia: diagnostic criteria for research studies. Report of the NINDS-AIREN International Workshop. Neurology..

[16] Garcia-Ptacek S, Cavallin L, Kåreholt I, Kramberger MG, Winblad B, Jelic V (2014). Subjective cognitive impairment subjects in our clinical practice. Dement Geriatr Cogn Dis Extra.

[17] Scheltens P, Leys D, Barkhof F, Huglo D, Weinstein HC, Vermersch P (1992). Atrophy of medial temporal lobes on MRI in “probable” Alzheimer’s disease and normal ageing: diagnostic value and neuropsychological correlates. J Neurol Neurosurg Psychiatry.

[18] Pasquier F, Leys D, Weerts J, Mounier-Vehier F, Barkhof F, Scheltens P (1996). Inter- and intraobserver reproducibility of cerebral atrophy assessment on MRI scans with hemispheric infarcts. Eur Neurol.

[19] Laforce R, Buteau JP, Paquet N, Verret L, Houde M, Bouchard RW (2010). The value of PET in mild cognitive impairment, typical and atypical/unclear dementias: A retrospective memory clinic study. Am J Alzheimers Dis Other Dement.

[20] Jagust W, Reed B, Mungas D, Ellis W, DeCarli C (2007). What does fluorodeoxyglucose PET imaging add to a clinical diagnosis of dementia?. Neurology..

[21] Sánchez-Juan P, Ghosh PM, Hagen J, Gesierich B, Henry M, Grinberg LT (2014). Practical utility of amyloid and FDG-PET in an academic dementia center. Neurology..

[22] Ossenkoppele R, Prins N, Pijnenburg Y, Lemstra A, van der Flier WM, Adriaanse S, Windhorst A (2013). Impact of molecular imaging on the diagnostic process in a memory clinic. Alzheimers Dement.

[23] Hellwig S, Frings L, Bormann T, Vach W, Buchert R, Meyer PT (2019). Amyloid imaging for differential diagnosis of dementia: incremental value compared to clinical diagnosis and [ 18 F]FDG PET. Eur J Nucl Med Mol Imaging.

[24] Leuzy A, Savitcheva I, Chiotis K, Lilja J, Andersen P, Bogdanovic N (2019). Clinical impact of [ 18 F]flutemetamol PET among memory clinic patients with an unclear diagnosis. Eur J Nucl Med Mol Imaging.

[25] Forsberg A, Engler H, Blomquist G, Långström B, Nordberg A (2012). The use of PIB-PET as a dual pathological and functional biomarker in AD. Biochim Biophys Acta.

[26] Perani D, Schillaci O, Padovani A, Nobili FM, Iaccarino L, Della Rosa PA, et al. Erratum to “A survey of FDG- and amyloid-PET imaging in dementia and GRADE analysis”. Biomed Res Int. 2014. 10.1155/2014/785039 PMC3977528 24772437

[27] Landau S, Harvey D, Madison C, Koeppe R, Reiman E, Foster N (2011). Associations between cognitive, functional, and FDG-PET measures of decline in AD and MCI. Neurobiol Aging.

[28] Herholz K, Westwood S, Haense C, Dunn G (2011). Evaluation of a calibrated 18F-FDG PET score as a biomarker for progression in alzheimer disease and mild cognitive impairment. J Nucl Med.

[29] Ito K, Fukuyama H, Senda M, Ishii K, Maeda K, Yamamoto Y (2015). Prediction of outcomes in mild cognitive impairment by using 18F-FDG-PET: A multicenter study. J Alzheimers Dis.

[30] Morbelli S, Brugnolo A, Bossert I, Buschiazzo A, Frisoni G, Galluzzi S (2015). Visual versus semi-quantitative analysis of 18F-FDG-PET in amnestic MCI: an European Alzheimer’s Disease Consortium (EADC) project. J Alzheimers Dis.

[31] Mosconi L, Perani D, Sorbi S, Herholz K, Nacmias B, Holthoff V (2004). MCI conversion to dementia and the APOE genotype: a prediction study with FDG-PET. Neurology..

[32] Toussaint P, Perlbarg V, Bellec P, Desarnaud S, Lacomblez L, Doyon J (2012). Resting state FDG-PET functional connectivity as an early biomarker of Alzheimer’s disease using conjoint univariate and independent component analyses. Neuroimage..

[33] Young J, Modat M, Cardoso MJ, Mendelson A, Cash D, Ourselin S (2013). Accurate multimodal probabilistic prediction of conversion to Alzheimer’s disease in patients with mild cognitive impairment. NeuroImage Clin.

[34] Choo I, Ni R, Schöll M, Wall A, Almkvist O, Nordberg A (2013). Combination of 18F-FDG PET and cerebrospinal fluid biomarkers as a better predictor of the progression to Alzheimer’s disease in mild cognitive impairment patients. J Alzheimers Dis.

[35] Arbizu J, Prieto E, Martínez-Lage P, Martí-Climent JM, García-Granero M, Lamet I, Pastor P, Riverol M (2013). Automated analysis of FDG PET as a tool for single-subject probabilistic prediction and detection of Alzheimer’s disease dementia. Eur J Nucl Med Mol Imaging.

[36] Cabral C, Morgado P, Campos Costa D, Silveira M (2015). Predicting conversion from MCI to AD with FDG-PET brain images at different prodromal stages. Comput Biol Med.

[37] Chételat G, Desgranges B, de la Sayette V, Viader F, Eustache F, Baron J (2003). Mild cognitive impairment: can FDG-PET predict who is to rapidly convert to Alzheimer’s disease?. Neurology..

[38] Drzezga A, Grimmer T, Riemenschneider M, Lautenschlager N, Siebner H, Alexopoulus P (2005). Prediction of individual clinical outcome in MCI by means of genetic assessment and18F-FDG PET. J Nucl Med.

[39] Gray KR, Wolz R, Heckemann RA, Aljabar P, Hammers A, Rueckert D (2012). Multi-region analysis of longitudinal FDG-PET for the classification of Alzheimer’s disease. Neuroimage..

[40] Perani D, Della Rosa PA, Cerami C, Gallivanone F, Fallanca F, Vanoli EG (2014). Validation of an optimized SPM procedure for FDG-PET in dementia diagnosis in a clinical setting. NeuroImage Clin.

[41] Caminiti SP, Ballarini T, Sala A, Cerami C, Presotto L, Santangelo R (2018). FDG-PET and CSF biomarker accuracy in prediction of conversion to different dementias in a large multicentre MCI cohort. NeuroImage Clin.

[42] Shaffer JL, Petrella JR, Sheldon FC, Choudhury KR, Calhoun VD, Coleman RE (2013). Predicting cognitive decline in subjects at risk for Alzheimer disease by using combined cerebrospinal fluid, MR imaging, and PET biomarkers. Radiology..

[43] Landau S, Harvey D, Madison C, Reiman E, Foster N, Aisen P (2010). Comparing predictors of conversion and decline in mild cognitive impairment. Neurology..

[44] Iaccarino L, Chiotis K, Alongi P, Almkvist O, Wall A, Cerami C (2017). A cross-validation of FDG-and amyloid-PET biomarkers in mild cognitive impairment for the risk prediction to dementia due to Alzheimer’s disease in a clinical setting. J Alzheimers Dis.

[45] Boccardi M, Altomare D, Ferrari C, Festari C, Guerra UP, Paghera B (2016). Assessment of the incremental diagnostic value of florbetapir F 18 imaging in patients with cognitive impairment: the incremental diagnostic value of amyloid PET with [18F]-florbetapir (INDIA-FBP) study. JAMA Neurol.

[46] Ramusino MC, Garibotto V, Bacchin R, Altomare D, Dodich A, Assal F (2020). Incremental value of amyloid-PET versus CSF in the diagnosis of Alzheimer’s disease. Eur J Nucl Med Mol Imaging.

[47] Rice L, Bisdas S (2017). The diagnostic value of FDG and amyloid PET in Alzheimer’s disease—a systematic review. Eur J Radiol.

