# Supplementary material for: Clinical impact of 18F-FDG-PET among memory clinic patients with uncertain diagnosis
Source: Eur J Nucl Med Mol Imaging. 2020 Jul 31;48(2):612–22. doi: 10.1007/s00259-020-04969-7 (PMC7835147; doi:10.1007/s00259-020-04969-7)
Supplement: Supplementary file 1 — (DOCX 19 kb) [file 259_2020_4969_MOESM1_ESM.docx]

**Clinical impact of ^18^F-FDG-PET among memory clinic patients with uncertain diagnosis.**

**European Journal of Nuclear Medicine and Molecular Imaging**

**Authors:** Giulia Perini^1,2^, Elena Rodriguez-Vieitez^1^, Ahmadul Kadir^3^, Arianna Sala^1^, Irina Savitcheva^4^, Agneta Nordberg^1,3^

^1^ Department of Neurobiology, Care Sciences and Society, Division of Clinical Geriatrics, Center for Alzheimer Research, Karolinska Institutet, 141 52 Huddinge, Stockholm, Sweden

^2^ Center for Cognitive and Behavioral Disorders, IRCCS Mondino Foundation and Dept of Brain and Behavior, University of Pavia, 27100, Pavia, Italy

^3^ Theme Aging, The Aging Brain Unit, Karolinska University Hospital, 141 86 Stockholm, Sweden

^4^ Department of Medical Radiation Physics and Nuclear Medicine Imaging, Karolinska University Hospital, Stockholm, Sweden

**Corresponding author**: Agneta Nordberg, MD, PhD, Professor, Karolinska Institutet, Department of Neurobiology, Care Sciences and Society, Division of Clinical Geriatrics, Karolinska Institutet, Stockholm, Sweden.

Telephone: +46 8 524 835 32; E-mail address: [Agneta.K.Nordberg@ki.se](mailto:Agneta.K.Nordberg@ki.se)

**ESM 1** Biomarkers measures in the group of patients with MCI at baseline (n=177), grouped by their follow-up diagnosis

|  | Follow-up diagnostic groups for n=177 patients diagnosed with MCI at baseline | | | | | |
| --- | --- | --- | --- | --- | --- | --- |
| Biomarkers measures | Stable  MCI  (n=75) | AD converters  (n=70) | FTLD converters  (n=18) | DLB converters  (n=4) | Dem NOS converters  (n=5) | Other Dem converters  (n=5) |
| FDG-PET, N. (%)  Negative^a^  Slight abnormalities^a^  Possible/probable AD^b^  Possible/probable FTLD^c^  Possible/probable DLB^d^  Wide spread hypometabolism | 6 (8.0)  37 (49.3)  11 (14.7)  19 (25.3)  0  2 (2.7) | 0  15 (21.4)  43 (61.4)  4 (5.7)  0  8 (11.4) | 0  2 (11.1)  2 (11.1)  14 (77.8)  0  0 | 0  0  2 (50.0)  0  2 (50.0)  0 | 0  2 (40.0)  1 (20.0)  1 (20.0)  0  1 (20.0) | 1 (20.0)  1 (20.0)  0  2 (40.0)  0  1 (20.0) |
| MTA, N. (%)  *N* of available data  0  1  2  3  4 | 66  13 (19.7)  28 (42.4)  15 (22.7)  7 (10.6)  3 (4.5) | 64  6 (9.4)  32 (50.0)  16 (25.0)  10 (15.6)  0 | 15  1 (6.7)  6 (40.0)  4 (26.7)  2 (13.3)  2 (13.3) | 4  0  0  4 (100)  0  0 | 4  1 (25.0)  0  2 (50.0)  1 (25.0)  0 | 5  1 (20.0)  3 (60.0)  0  1 (20.0)  0 |
| GCA, N. (%)  *N* of available data  0  1  2  3 | 54  8 (14.8)  25 (46.3)  18 (33.3)  3 (5.5) | 57  3 (5.3)  29 (50.9)  24 (42.1)  1 (1.7) | 14  1 (7.1)  5 (35.7)  5 (35.7)  3 (21.4) | 4  0  1 (25.0)  3 (75.0)  0 | 2  0  2 (100)  0  0 | 5  0  1 (20.0)  4 (80.0)  0 |
| CSF biomarkers, mean (sd), ng/L  *N* of available data  Aβ1-42^e^  p-tau^f^  t-tau^g^ | 59  902.1  (288.7)  50.1  (29.6)  306.3  (225.5) | 63  587.0  (130.4)  86.5  (62.8)  580.4  (260.7) | 15  1084.4 (379.2)  51.4  (26.0)  364.1  (302.5) | 3  690.3  (340.9)  31.3  (7.6)  205.3  (42.2) | 4  956.5  (493.6)  40.2  (23.2)  331.7  (151.1) | 4  810.0  (253.4)  49.7  (21.1)  336.5  (214.0) |
| [18F]Flutemetamol^h^, N. (%)  *N* of available data  Positive  Negative | 14  5 (35.7)  9 (64.3) | 21  21 (100)  0 | 1  0  1 (100) | 1  0  1 (100) | 2  0  2 (100) | 2  0  2 (100) |

*AD*, Alzheimer’s disease; *Dem NOS*, dementia not otherwise specified; *DLB*, dementia with Lewy bodies; *FTLD*, frontotemporal lobar degeneration; *GCA*, global cortical atrophy; *MCI*, mild cognitive impairment; *MTA*, medial temporal atrophy

Wilcoxon and Fisher tests (with Bonferroni post-hoc correction) (excluding ‘Other Dem’ converters group)

^a^ FDG negative/slight abnormalities: stable MCI > AD converters and FTLD converters (p <0.001, 0.01)

^b^ FDG possible/probable AD: AD converters > stable MCI, FTLD converters (p <0.001, 0.01)

^c^ FDG possible/probable FTLD: FTLD converters > stable MCI, AD converters (p <0.001), stable MCI > AD converters (p <0.05)

^d^ FDG possible/probable DLB: DLB converters > stable MCI, AD converters (p <0.05)

^e^ Aβ42: AD converters < stable MCI and FTLD converters (p <0.001)

^f^ p-tau: AD converters > stable MCI and FTLD converters (p <0.001, 0.01)

^g^ t-tau: AD converters > stable MCI and FTLD converters (p <0.001, 0.01)

^h^ [18]Flutemetamol: AD converters > stable MCI and Dem NOS converters (p <0.001, 0.05)
