# Supplementary material for: Clinical impact of 18F-FDG-PET among memory clinic patients with uncertain diagnosis
Source: Eur J Nucl Med Mol Imaging. 2020 Jul 31;48(2):612–22. doi: 10.1007/s00259-020-04969-7 (PMC7835147; doi:10.1007/s00259-020-04969-7)
Supplement: Supplementary file 2 — (DOCX 18 kb) [file 259_2020_4969_MOESM2_ESM.docx]

**Clinical impact of ^18^F-FDG-PET among memory clinic patients with uncertain diagnosis.**

**European Journal of Nuclear Medicine and Molecular Imaging**

**Authors:** Giulia Perini^1,2^, Elena Rodriguez-Vieitez^1^, Ahmadul Kadir^3^, Arianna Sala^1^, Irina Savitcheva^4^, Agneta Nordberg^1,3^

^1^ Department of Neurobiology, Care Sciences and Society, Division of Clinical Geriatrics, Center for Alzheimer Research, Karolinska Institutet, 141 52 Huddinge, Stockholm, Sweden

^2^ Center for Cognitive and Behavioral Disorders, IRCCS Mondino Foundation and Dept of Brain and Behavior, University of Pavia, 27100, Pavia, Italy

^3^ Theme Aging, The Aging Brain Unit, Karolinska University Hospital, 141 86 Stockholm, Sweden

^4^ Department of Medical Radiation Physics and Nuclear Medicine Imaging, Karolinska University Hospital, Stockholm, Sweden

**Corresponding author**: Agneta Nordberg, MD, PhD, Professor, Karolinska Institutet, Department of Neurobiology, Care Sciences and Society, Division of Clinical Geriatrics, Karolinska Institutet, Stockholm, Sweden.

Telephone: +46 8 524 835 32; E-mail address: [Agneta.K.Nordberg@ki.se](mailto:Agneta.K.Nordberg@ki.se)

**ESM 2** Biomarkers measures in the group of patients with diagnosis of dementia at baseline (n=100), grouped by their follow-up diagnosis

|  | Follow-up diagnostic groups for n=100 patients diagnosed with dementia at baseline | | | | |
| --- | --- | --- | --- | --- | --- |
| Biomarkers measures | AD  (n=45) | FTLD  (n=27) | DLB  (n=16) | Dem NOS  (n=3) | Other Dem  (n=9) |
| FDG-PET, N. (%)  Negative  Slight abnormalities  Possible/probable AD^a^  Possible/probable FTLD^b^  Possible/probable DLB^c^  Wide spread hypometabolism | 0  7 (15.5)  34 (75.5)  2 (4.4)  1 (2.2)  1 (2.2) | 0  1 (3.7)  2 (7.4)  22 (81.5)  1 (3.7)  1 (3.7) | 0  0  1 (6.2)  1 (6.2)  12 (75.0)  2 (12.5) | 0  1 (33.3)  0  1 (33.3)  0  1 (33.3) | 1 (11.1)  2 (22.2)  0  3 (33.3)  2 (22.2)  1 (14.3) |
| MTA, N. (%)  *N* of available data  0  1  2  3  4 | 42  0  15 (35.7)  18 (42.8)  8 (19.0)  1 (2.4) | 19  0  6 (31.6)  9 (47.4)  3 (15.8)  1 (5.3) | 14  0  5 (35.7)  7 (50.0)  2 (14.3)  0 | 3  1 (33.3)  0  0  0  2 (66.7) | 7  1 (14.3)  4 (57.1)  2 (28.6)  0  0 |
| GCA, N. (%)  *N* of available data  0  1  2  3 | 34  0  12 (35.3)  19 (55.9)  3 (8.8) | 18  0  7 (38.9)  8 (44.4)  3 (16.7) | 12  0  5 (41.7)  7 (58.3)  0 | 3  1 (33.3)  0  0  2 (66.7) | 7  0  5 (71.4)  2 (28.6)  0 |
| CSF biomarkers, mean (sd), ng/L  *N* of available data  Aβ1-42^d^  p-tau^e^    t-tau^f^ | 41  671.8  (316.6)  73.1  (34.1)  527.9  (318.6) | 24  1083.2  (322.3)  45.6  (20.7)  342.3  (170.4) | 13  866.8  (297.5)  43.9  (18.0)  284.0  (93.4) | 3  1116.0  (287.7)  56.3  (10.7)  380.7  (138.3) | 8  1115.7  (257.1)  38.2  (13.4)  218.1  (111.6) |
| [18F]Flutemetamol, N. (%)  *N* of available data  Positive  Negative | 6  5 (83.3)  1 (16.7) | 3  1 (33.3)  2 (66.7) | 0 | 0 | 2  0  2 (100) |

*AD*, Alzheimer’s disease; *Dem NOS*, dementia not otherwise specified; *DLB*, dementia with Lewy bodies; *FTLD*, frontotemporal lobar degeneration; *GCA*, global cortical atrophy; *MCI*, mild cognitive impairment; *MTA*, medial temporal atrophy

Wilcoxon and Fisher tests (with Bonferroni post-hoc correction) (excluding ‘Other Dem’ e ‘Dem NOS’ groups)

^a^ FDG possible/probable AD: AD > FTLD, DLB (p <0.001)

^b^ FDG possible/probable FTLD: FTLD > AD, DLB (p <0.001)

^c^ FDG possible/probable DLB: DLB > AD, FTLD (p <0.001)

^d^ Aβ42: AD < FTLD (p < 0.001)

^e^ p-tau: AD > FTLD and DLB (p < 0.001 and < 0.01)

^f^ t-tau: AD > DLB and FTLD (p < 0.01 and < 0.05)
