# Supplementary material for: Clinical impact of 18F-FDG-PET among memory clinic patients with uncertain diagnosis
Source: Eur J Nucl Med Mol Imaging. 2020 Jul 31;48(2):612–22. doi: 10.1007/s00259-020-04969-7 (PMC7835147; doi:10.1007/s00259-020-04969-7)
Supplement: Supplementary file 3 — (DOCX 23 kb) [file 259_2020_4969_MOESM3_ESM.docx]

**Clinical impact of ^18^F-FDG-PET among memory clinic patients with uncertain diagnosis.**

**European Journal of Nuclear Medicine and Molecular Imaging**

**Authors:** Giulia Perini^1,2^, Elena Rodriguez-Vieitez^1^, Ahmadul Kadir^3^, Arianna Sala^1^, Irina Savitcheva^4^, Agneta Nordberg^1,3^

^1^ Department of Neurobiology, Care Sciences and Society, Division of Clinical Geriatrics, Center for Alzheimer Research, Karolinska Institutet, 141 52 Huddinge, Stockholm, Sweden

^2^ Center for Cognitive and Behavioral Disorders, IRCCS Mondino Foundation and Dept of Brain and Behavior, University of Pavia, 27100, Pavia, Italy

^3^ Theme Aging, The Aging Brain Unit, Karolinska University Hospital, 141 86 Stockholm, Sweden

^4^ Department of Medical Radiation Physics and Nuclear Medicine Imaging, Karolinska University Hospital, Stockholm, Sweden

**Corresponding author**: Agneta Nordberg, MD, PhD, Professor, Karolinska Institutet, Department of Neurobiology, Care Sciences and Society, Division of Clinical Geriatrics, Karolinska Institutet, Stockholm, Sweden.

Telephone: +46 8 524 835 32; E-mail address: [Agneta.K.Nordberg@ki.se](mailto:Agneta.K.Nordberg@ki.se)

**ESM 3** Analyses of 266 subjects, excluding 11 subjects who performed Flutemetamol PET prior to post-FDG-PET diagnosis

Clinical impact of FDG-PET in terms of changes in diagnosis from pre- to post-FDG-PET imaging, according to FDG-PET classification

| Diagnosis pre-FDG-PET | Change in diagnosis post- vs pre-FDG-PET | FDG-PET classification | Number of  those with change | Diagnosis post-FDG-PET | Final diagnosis |
| --- | --- | --- | --- | --- | --- |
| MCI (n=170) | 53/170 (31%) | slight abnormalities | 8 | 5 AD, 1 FTLD, 1 Dem NOS, 1 Other | 4 AD, 1 FTLD, 2 Dem NOS, 1 Other |
|  |  | possible AD | 9 | 8 AD, 1 Other | 6 AD, 1 FTLD, 2 MCI |
|  |  | probable AD | 18 | 16 AD, 2 Dem NOS | 16 AD, 1 FTLD, 1 DLB |
|  |  | possible FTLD | 8 | 4 FTLD, 1 AD, 3 Dem NOS | 4 FTLD, 2 AD, 1 Dem NOS, 1 Other |
|  |  | probable FTLD | 2 | 2 FTLD | 2 FTLD |
|  |  | probable DLB | 1 | 1 DLB | 1 DLB |
|  |  | wide spread hypometabolism | 7 | 5 AD, 1 FTLD, 1 Other | 6 AD, 1 Other |
| AD (n=33) | 3/33 (9%) | probable AD | 2 | 1 FTLD, 1 DLB | 1 FTLD, 1 DLB |
|  |  | possible FTLD | 1 | 1 FTLD | 1 FTLD |
| FTLD (n=14) | 0/14 (0%) |  |  |  |  |
| DLB (n=10) | 1/10 (10%) | probable AD | 1 | 1 AD | 1 AD |
| Dem NOS (n=29) | 18/29 (62%) | slight abnormalities | 1 | 1 AD | 1 AD |
|  |  | possible AD | 1 | 1 AD | 1 AD |
|  |  | probable AD | 7 | 7 AD | 7 AD |
|  |  | possible FTLD | 2 | 2 AD | 1 FTLD, 1 AD |
|  |  | probable FTLD | 5 | 5 FTLD | 5 FTLD |
|  |  | probable DLB | 1 | 1 DLB | 1 DLB |
|  |  | wide spread hypometabolism | 1 | 1 Other | 1 Other |
| Other (n=10) | 5/10 (50%) | probable FTLD | 1 | 1 FTLD | 1 FTLD |
|  |  | possible FTLD | 2 | 1 FTLD, 1 DLB | 1 FTLD, 1 DLB |
|  |  | possible DLB | 1 | 1 DLB | 1 DLB |
|  |  | probable DLB | 1 | 1 DLB | 1 DLB |

*AD*, Alzheimer’s disease; *Dem NOS*, dementia not otherwise specified; *DLB*, dementia with Lewy bodies; *FTLD*, frontotemporal lobar degeneration

Incremental diagnostic value of FDG-PET, in terms of comparing each of the pre- and post-FDG-PET accuracy to the final follow-up diagnosis as the reference, in the group of patients with uncertain dementia diagnoses at baseline (n=96)

|  | diagnosis AD vs non-AD | | diagnosis FTLD vs non-FTLD | | diagnosis DLB vs non-DLB | |
| --- | --- | --- | --- | --- | --- | --- |
|  | pre-FDG-PET | post-FDG-PET | pre-FDG-PET | post-FDG-PET | pre-FDG-PET | post-FDG-PET |
| SE (%) | 63 | 88 | 48 | 82 | 56 | 88 |
| SP (%) | 89 | 93 | 99 | 99 | 99 | 100 |
| ACC (%)^a^ | 77 | 91 | 84 | 94 | 92 | 98 |
| LR+ | 5.6 | 11.8 | 34.4 | 58.2 | 46.9 | infinity |
| LR- | 0.4 | 0.1 | 0.5 | 0.2 | 0.4 | 0.1 |

*ACC*, accuracy; *AD*, Alzheimer’s disease; *DLB*, dementia with Lewy bodies; *FTLD*, frontotemporal lobar degeneration; *LR*, likelihood-ratio; *SE*, sensitivity; *SP*, specificity

^a^ McNemar's test

-Accuracy of AD vs non AD diagnosis improved after FDG-PET (p <0.01)

Accuracy of FTLD vs non FTLD diagnosis improved after FDG-PET (p <0.01)

Accuracy of DLB vs non DLB diagnosis improved after FDG-PET (p <0.05)

FDG-PET classification accuracy in detecting AD and FTLD converters, in the group of patients with MCI at baseline (n=170) and in detecting AD, FTLD and DLB, in the group of patients with uncertain dementia diagnoses at baseline (n=96)

|  | FDG-PET in MCI subjects at baseline (n=170) | | FDG-PET in subjects with uncertain dementia diagnoses at baseline (n=96) | | |
| --- | --- | --- | --- | --- | --- |
|  | AD vs non-AD | FTLD vs non-FTLD | AD vs non-AD | FTLD vs non-FTLD | DLB vs non-DLB |
| SE (%) | 64 | 78 | 77 | 82 | 75 |
| SP (%) | 86 | 84 | 94 | 90 | 96 |
| ACC (%) | 77 | 83 | 87 | 88 | 93 |
| LR+ | 4.4 | 4.7 | 13.5 | 8.1 | 20.3 |
| LR- | 0.4 | 0.3 | 0.2 | 0.2 | 0.3 |

*ACC*, accuracy; *AD*, Alzheimer’s disease; *DLB*, dementia with Lewy bodies; *FTLD*, frontotemporal lobar degeneration; *LR*, likelihood-ratio; *SE*, sensitivity; *SP*, specificity

Biomarkers accuracy in detecting AD converters, in the group of patients with MCI at baseline (n=170)

|  | AD converters vs non-AD converters | | | | |
| --- | --- | --- | --- | --- | --- |
|  | FDG-PET | Aβ1-42 | p-tau | t-tau | Flutemetamol-PET |
| SE (%) | 64 | 42 | 44 | 73 | 100 |
| SP (%) | 86 | 90 | 86 | 74 | 82 |
| ACC (%) | 77 | 70 | 68 | 73 | 91 |
| LR+ | 4.4 | 4.4 | 3.0 | 2.8 | 5.7 |
| LR- | 0.4 | 0.6 | 0.7 | 0.4 | 0 |

*ACC*, accuracy; *AD*, Alzheimer’s disease; *LR*, likelihood-ratio; *SE*, sensitivity; *SP*, specificity
